# The Role of IL-17 in Protection against Mucosal *Candida* Infections

**DOI:** 10.3390/jof3040052

**Published:** 2017-09-27

**Authors:** Bemnet G. Mengesha, Heather R. Conti

**Affiliations:** Department of Biological Sciences, The University of Toledo, 2801 West Bancroft St., Toledo, OH 43606, USA; bemnet.mengesha@rockets.utoledo.edu

**Keywords:** *C. albicans*, mucosal fungal infections, oropharyngeal candidiasis, chronic mucocutaneous candidiasis

## Abstract

Interleukin-17 (IL-17) is a proinflammatory cytokine produced by adaptive CD4+ T helper cells and innate lymphocytes, such as γδ-T cells and TCRβ+ “natural” Th17 cells. IL-17 activates signaling through the IL-17 receptor, which induces other proinflammatory cytokines, antimicrobial peptides and neutrophil chemokines that are important for antifungal activity. The importance of IL-17 in protective antifungal immunity is evident in mice and humans, where various genetic defects related to the IL-17-signaling pathway render them highly susceptible to forms of candidiasis such oropharyngeal candidiasis (OPC) or more broadly chronic mucocutaneous candidiasis (CMC), both caused mainly by the opportunistic fungal pathogen *Candida albicans*. OPC is common in infants and the elderly, HIV/AIDS and patients receiving chemotherapy and/or radiotherapy for head and neck cancers. This review focuses on the role of IL-17 in protection against candidiasis, and includes a brief discussion of non-*Candida albicans* fungal infections, as well as how therapeutic interventions blocking IL-17-related components can affect antifungal immunity.

## 1. Introduction

The incidence of fungal infections is increasing worldwide due in part to the growing populations of immunodeficient individuals, which poses an increasing threat to human health. Various immunocompromising conditions such as HIV/AIDS [1,2,3], chemotherapy, radiotherapy and antibiotic use [4], as well as congenital immune defects [5] have been implicated in host susceptibility to fungal infections, especially chronic mucocutaneous candidiasis. *Candida* species are part of the normal microflora of the gastrointestinal and reproductive tracts of about 50–80% of healthy individuals [6,7], but can become pathogenic in immunocompromised patients [1]. Due to the increasing incidence of fungal infections, combined with the lack of effective vaccines and drug therapies, fungal diseases have emerged as a significant cause of morbidity and mortality [8], suggesting the need for more effective anti-fungal therapies. IL-17 and Th17 cells are critical for broad immunity to extracellular microbes, and recent studies in both humans and animal models have elucidated the overwhelming role of the Th17/IL-17 axis in protection against superficial candidiasis, caused mainly by *Candida albicans*, which is the primary focus of this review. 

Four main genera of fungi, *Candida*, *Cryptococcus*, *Aspergillus* and *Pneumocystis*, cause more than 90% of the mortality due to fungal infections. [4]. Several non-*albicans* species of *Candida* (*C. glabrata*, *C.tropicalis*, *C. parapsilosis*, *C. krusei* and *C. dubliniensis*) cause disease, yet *C. albicans* is the main causative agent and the best characterized species. Multiple manifestations of *Candida* infection can occur when anti-fungal immunity is defective. Mucocutaneous forms of disease include oral/oropharyngeal (OPC, thrush), vaginal and cutaneous candidiasis [6,9]. Disseminated infection, or candidemia, is the fourth most common nosocomial infection with a high mortality rate of 40–60% [10]. OPC is one of the most common opportunistic fungal infections in humans, and can severely impact the quality of life for immunocompromised populations [3,9]. Vulvovaginal candidiasis (VVC) is diagnosed in 75% of women of reproductive age, and recurrent VVC can significantly impact quality of life and lead to increased treatment costs [9]. 

Both innate and adaptive immunity play a role in protection against fungal infections, although the relative contribution of each can differ based on the anatomical location of disease [9]. For example, on mucosal surfaces innate immune responses play a major role in protection, which has been shown particularly well in mouse models of disease. Mice are naïve to *C. albicans,* therefore infection induces an acute response [11]. However, in humans adaptive immunity is essential for protection against mucosal candidiasis, indicated by the high susceptibility of HIV+ and T-cell deficient patients to disease [9]. In humans innate immunity is also important in defense against disseminated candidiasis. Although IL-17 is implicated in systemic or disseminated candidiasis, Th1 and natural killer (NK) cells, via IFN-γ, are critical which indicates tissue-specific immunity [12]. Overall, interleukin-17 (IL-17A)-mediated antifungal immunity is essential in oral and dermal candidiasis, and at various stages involves both hematopoietic and non-hematopoietic cells. Hematopoietic cells such as phagocytes (neutrophils and monocytes/macrophages), adaptive Th17 cells, natural (n) Th17 cells, dendritic cells (DC), non-major histocompatibility complex (non-MHC) restricted T-cells subsets such as γδ T-cell, as well as non-hematopoietic cells including mucosal epithelial cells participate in antifungal immunity. Epithelial cells (ECs) express the IL-17 receptor (IL-17RA/RC) and are involved in fungal clearance via production of various proinflammatory cytokines and antimicrobial peptides during infection [11]. The role of IL-17 in VVC is less clear though. Interestingly, patients with IL-17-defects do not show an increased susceptibility to VVC as they do to other mucocutaneous forms [13]. Also, one study showed a protective IL-17 response in an estrogen-induced model of VVC, while another demonstrated that IL-17 was not beneficial [14,15]. In addition, unlike in other mucosal forms of candidiasis, neutrophils are thought to be pathogenic in VVC [16]. Instead, protection against candidiasis in the vaginal tract involves antimicrobial peptides, but also relies on an intact epithelial layer including a balanced microbial flora and pH [17]. Further research is necessary to determine the role for IL-17-mediated immunity during VVC. 

## 2. Pattern Recognition of *Candida albicans*

Infections caused by *C. albicans* are the most frequent fungal infections on mucosal surfaces [1]. *C. albicans* is a dimorphic fungus which exists as a unicellular yeast and an invasive filamentous hyphal (pseudohyphal) form. *C. albicans* is maintained as a commensal on host epithelial surfaces, but can become pathogenic and potentially life threatening under invasive conditions. The morphological transition from yeast to hyphae is one of the most important virulence factors mediating pathogenesis as expression of other virulence genes varies depending on morphological stage [18]. Host recognition of either form of *C. albicans* via immunoreceptors is required in order to mount an appropriate immune response including activation of IL-17. 

Pathogen recognition by the host immune system broadly involves four classes of pattern recognition receptors (PRRs): Toll-like receptors (TLRs), C-type lectin receptors (CLRs), nucleotide-binding oligomerization domain-like (NOD-like) receptors (NLRs) and retinoic-acid-inducible gene I (RIG-I) like receptors (RLRs) [19]. PRRs recognize pathogen associated molecular patterns (PAMPs), which are conserved microbial components. Engagement of PRRs triggers intracellular signaling ultimately activating innate immunity through expression of various genes involved in inflammation and host defense [20]. 

Recently, great strides have been made in understanding pattern recognition of *Candida* and the signaling pathways initiated, and are the topics of comprehensive reviews including [21,22,23]. Briefly, the CLRs, especially dectin-1 and dectin-2, play a major role in innate recognition of fungal pathogens including *Candida* spp., although TLRs and NLRs also contribute to the sensing of *Candida* [21,22,23]. The cell wall of *C. albicans* consists of an outer layer of mannoproteins with *O*-glycosylated oligosaccharide and *N*-glycosylated polysaccharide moieties, with an inner layer of chitin and β (1, 3) and β (1, 6) glucans [19]. The CLRs (including dectin-1, -2, -3, Mincle and the mannose receptor) detect carbohydrate mannans and glucans [24]. Downstream signaling through these receptors activates NF-κB and other signaling pathways leading to pro-inflammatory responses including the production of Th17 inductive cytokines such as IL-6, IL-1β and IL-23, while suppressing IL-12p35 and thus Th1 differentiation [25,26,27]. Alvarez et al. have shown using zymosan, a fungal cell wall extract containing β-glucan moieties, the mechanism by which dectin-1 and TLR-2 signaling leads to transcriptional repression of *IL12A*, the gene encoding IL-12p35 [28,29]. Additionally, fungal epitopes activate STAT3 (necessary for Th17 proliferation and function, see below) via secondary mediators which insures initial pattern recognition of *Candida* leads to a cytokine environment poised to activate a Th17 response [30]. Dectin-1 plays a significant role in mediating the cellular response to *Candida*, and is activated during budding or the hyphal transition, when the inner glucan layer is accessible [22]. In this way, morphotype switching also allows *Candida* to evade the host immune response through the shielding of glucans from dectin-1 recognition [31]. The specific morphotypes also differentially induce an adaptive Th response, and potentially promote the transition from health to pathogenicity. Initial in vitro studies showed that *C. albicans* hyphae prime Th17 cells, while yeast cells induce a Th1 response [32]. In contrast, the Kaplan group demonstrated that yeast induce Th17 differentiation in a dectin-1-dependent manner, yet hyphal form induces Th1 cells independently of dectin-1 during systemic candidiasis [33]. 

Dectin-2 recognizes α-mannans, and is expressed by macrophages, dendritic cells and neutrophils. Dectin-2 initiates a Th17-skewed response to *Candida*, and is essential for antifungal defense against *Candida* [34,35]. Interestingly, Dectin2^−/−^ mice are susceptible to disseminated candidiasis caused by both *C. albicans* and *C. glabrata*, indicating a role for Th17/IL-17-mediated immunity to non-albicans-*Candida* spp. [36]. 

## 3. IL-17 Mediated Anti-Fungal Immunity

The IL-17 family consists of six cytokines (IL-17A—IL-17F) and five receptors (IL-17RA—IL-17RE). IL-17A (IL-17) and IL-17F form homo- and heterodimers, and signal through a receptor complex (IL-17R) consisting of IL-17RA and IL-17RC subunits. The IL-17A/IL-17R signaling axis has have been studied most extensively in the context of candidiasis (reviewed in [37]). Tissue distribution analysis using various murine cell lines shows ubiquitous distribution of IL-17RA in both hematopoietic and non-hematopoietic cell types, while IL-17RC expression limits IL-17 signaling to non-hematopoietic tissue including mesenchymal, epithelial and endothelial cells [38]. In this way IL-17-signaling has been especially important in mucosal forms of candidiasis. 

IL-17 is involved as a key regulator of antifungal immunity through induction of a signature gene profile including pro-inflammatory cytokines, antimicrobial peptides and chemokines [39]. When IL-17 binds to IL-17RA/RC, signaling mediators such as Act1 and TRAF6 are recruited to the receptor, which leads to downstream activation of NF-κB, MAPK and C/EBP pathways. The importance of IL-17-signaling in anti-*Candida* defense is indicated by the high susceptibility of IL-17RA^−/−^ mice to OPC, which correlates with defects in neutrophil recruitment and reduced antimicrobial peptide (AMP) production [40]. Gene profiling studies of oral mucosa from WT and IL-17RA^−/−^ mice have also helped to elucidate IL-17RA-regulated gene targets such as those encoding pro-inflammatory cytokines, chemokines and antimicrobial peptides. IL-17RA^−/−^ mice show reduced levels of neutrophil-recruiting *CXC* chemokines and growth factors [41], including reduced levels of *CXCL*1, *CXCL*5 and granulocyte colony-stimulating factor (G-CSF) [42]. 

Neutrophils are an important component of the mammalian innate immune system, and are one of the first cells to traffic into the site of infection. Neutrophils battle against invading fungi and bacteria through various antimicrobial defense mechanisms [18,43]. It is well established that neutrophils are essential for defense against disseminated candidiasis in both humans, as well as mice, where neutrophil-depleted mice failed to control *C. albicans* dissemination [44,45,46]. However, the role of neutrophils in protection against OPC is less clear. Patients with isolated neutropenia are not normally susceptible to OPC [47,48,49]. In addition, while neutrophil depleted mice are susceptible to OPC, the contribution of IL-23/IL-17-mediated regulation of the neutrophil response is not completely understood [41]. Despite the assumption that neutrophil infiltration is controlled by IL-17RA in OPC [37], recent findings show IL-1R signaling regulates fungal clearance via infiltration of neutrophils to the infection site [50]. Further studies of mice deficient in IL-17 and IL-23 via antibody-depletion also suggested the presence of IL-17RA-independent mechanisms of neutrophil recruitment in mucosal immunity against *Candida* infection [41]. Our recent finding centered on *Candida* infection in oral epithelium showed neutrophil influx to the infection site is not exclusively modulated by IL-17RA, implying the involvement of additional genes [51]. Further understanding of the protective and/or pathogenic nature of neutrophils in mucosal infection will be aided by unique model systems such as a zebrafish swim bladder candidiasis model, which allows in vivo tracking of host-pathogen interactions in a highly efficient visual manner [52]. 

In addition to contributing to the neutrophil response, IL-17 also regulates production of various antimicrobial peptides including defensins (β-defensins, BDs), calprotectin (S100A8/9) and mucins. Murine β-defensin 3 (mBD3, functional homologue of human BD2) has been shown to be involved in protection against OPC, and is strongly IL-17-dependent [51]. In addition to its antimicrobial properties, mBD3 is also a chemoattractant. Similar to the chemokine CCL20, mBD3 is a ligand for the chemokine receptor CCR6, which is expressed by various IL-17 producing lymphocytes [53]. In this way, IL-17 may prove to function in immune modulation through the recruitment of additional IL-17+ lymphocytes to the site of infection, although this has not been confirmed in vivo. IL-17 also stimulates expression of mucin gene *MUC5B* which is involved in *Candida* clearance by suppressing virulence factors such as genes related to adhesion, filamentation and biofilm formation in oral epithelium [54]. In all, signaling through the IL-17 receptor results in a neutrophil influx, which is protective depending on anatomical location of disease, as well as induction of antimicrobial proteins which cooperate for fungal control. 

## 4. Innate and Adaptive Sources of IL-17 in Candidiasis

Adaptive CD4+ T helper 17 (Th17) cells arise from naïve precursors through signals from various inductive cytokines including IL-6, IL1β, TGF-β and IL-23. Moreover, the transcription factors retinoic acid-related orphan receptor gamma (RORγt) and signal transducer and activator of transcription-3 (STAT3) are involved in the differentiation and function of Th17 cells [32,55]. Th17 cells produce IL-17 (IL-17A), IL-17F, IL-21, IL-22 and GM-CSF. The importance of CD4+ T cells in antifungal immunity was first indicated by the increased sensitivity of HIV/AIDS patients to OPC [56]. More recently it was shown that human *Candida*-specific memory T cells are predominantly Th17 cells [32,57]. 

Before the discovery of Th17 cells, Th1 and IL-12 were thought to mediate the main protective mechanisms in candidiasis. This conclusion was reached, in part, based on the susceptibility of IL-12p40^−/−^ mice to OPC, yet these mice are deficient in both IL-12 and IL-23 because these cytokines share the p40 subunit [9]. Recently, more specific studies using IL-12p35^−/−^ (Th1-deficient) and IL-23p19^−/−^ (Th17-deficient) mice uncovered a role for Th17 in antifungal immunity, whereas Th17-deficient mice were highly susceptible to OPC and Th1-deficient mice were not [42]. Since IL-23 is essential for the proliferation and function of both adaptive and innate sources of IL-17, mice deficient in IL-23 lack a proper IL-17 response and therefore show high oral fungal burden during OPC, which is in contrast to IL-12p35 deficient mice that remain healthy during OPC. This newer finding also aligned well with the resistance of mice deficient in IFN-γ (a Th1-related cytokine) to OPC [58]. Additionally, mice lacking downstream IL-17-signaling components (IL-17RA^−/−^, IL-17RC^−/−^ or Act1^−/−^) are highly susceptible OPC [11,42,59]. The Th17 produced cytokine IL-22 is also important in antifungal immune responses, yet in experimental OPC IL-22^−/−^ mice are only mildly susceptible to OPC compared to IL-17RA^−/−^ mice [42]. 

In addition to Th17 cells there are important innate cellular sources of IL-17, termed “Type 17” cells. Type 17 cells are dependent on IL-23 and RORγt expression for function and IL-17 production. Type 17 subsets include γδ-T, nTh17, natural killer T (NKT), lymphoid tissue inducer (LTi) and group 3 innate lymphoid cells (ILCs) [53]. Neutrophils also produce IL-17, but not in response to *Candida* [41]. In adults, adaptive Th17 cells are a critical component of the protective response against candidiasis, yet in naive mice and presumably infants an effective immune response that clears OPC is mounted at early time points before adaptive immunity normally functions [37]. Accordingly, γδ-T and nTh17 cells are major innate sources of IL-17 during acute OPC [37]. A role for group 3 ILCs has been reported, but the finding remains controversial as Rag1^−/−^ mice with intact ILCs are susceptible to OPC (reviewed in [60]).

## 5. Studying IL-17 Deficiencies in Murine and Human Candidiasis

*C. albicans* is not a commensal in mice which has allowed the design of candidiasis models that recapitulate the initial exposure and innate immune response evident in infants, also naïve to *Candida*, in which OPC is usually self-limiting. Subsequent development of re-challenge models of adaptive responses to candidiasis, along with these acute models, has allowed elucidation of the importance of IL-17 in protection against OPC [61]. Oral candidiasis models standardly establish mucosal infection through immunosuppression using corticosteroids, and have been used extensively to study *C. albicans* virulence factors [62]. Other studies using gene knockout mice deficient in Th17/IL-17 pathway components (which do not require further immunosuppression with cortisone beyond the genetic defect) align with findings showing deficiencies related to IL-17 lead to increased susceptibility to chronic mucocutaneous candidiasis in humans. 

Newly described primary immune defects in humans along the IL-17/Th17 pathway lead to increased susceptibility to candidiasis [63,64]. Genetic polymorphisms which potentially lead to defects in *Candida* recognition (*CARD9*, *DECTIN1*), Th17 differentiation and proliferation (*STAT3*, *STAT1*, *TYK2*, *IL-12B*, *IL-12RB1)* or IL-17R signaling (*IL-17RA*, *IL-17RC, ACT1, IL-17F)* can increase susceptibility to CMC, and lend evidence for the importance of the Th17/IL-17axis in antifungal immunity [65]. 

*DECTIN1* defects can lead to an increased predisposition to *Candida* infections, especially in hematopoietic stem cell transplant patients [66,67]. Deficiencies in *Candida* detection pathways are not straightforward though, as patients with defects in *DECTIN1* are common in a large proportion of the population without an increased predisposition to fungal infections [13,68]. Additionally, *CARD9* defects, while rare, lead to systemic candidiasis with potential central nervous system (CNS) involvement due to CARD9 expression on monocytes and macrophages, rather than mucosal forms of disease [69,70,71]. Intriguingly, patients with CARD9 deficiencies also develop invasive extrapulmonary aspergillosis caused by *Aspergillus*, further supporting the importance of triggering a Th17 response following other fungal infections [72]. 

Hyper-IgE syndrome illustrates the importance of intact STAT3 signaling pathways for Th17 differentiation and proliferation, and shows how defects in this pathway lead to increased fungal susceptibility. HIES is caused by dominant-negative mutations in the DNA-binding or SH2 domains of STAT3 that lead to Th17/IL-17-related defects [37]. STAT3 is down stream of IL-6, IL-21 and IL-23, which are necessary for Th17 differentiation. Mutations in STAT3 lead to a significant deficiency in Th17 and patients exhibit increased susceptibility to OPC and mucocutaneous candidiasis due to deficiencies in antimicrobial peptides such as β-defensins and histatins [73,74]. Individuals with STAT1 gain-of-function mutations are also susceptible to CMC, yet these mutations are also linked to the Th17 pathway and IL-17 immunity [75]. Th17 differentiation is inhibited by cytokines such as IL-27, IFN-γ and IFN-α/β, which are all upstream of STAT1 [76,77,78]. These mutations lead to decreased Th17 cell frequencies and a predisposition to CMC, even though there is evidence type I interferons protect against candidiasis [79]. Nonetheless, perturbations in differentiation or proliferation of Th17 cells can lead to an increased incidence of candidiasis. 

Deficiencies in IL-17-signaling components that lead to increased susceptibility to CMC offer convincing evidence for the importance of the IL-17 pathway in protection against fungal infections [80]. Individuals with mutations in the thymic transcription factor *AIRE*, involved in central tolerance, produce anti-IL-17 and anti-IL-22 autoantibodies and are susceptible to CMC through neutralization of Th17-related cytokines [81,82]. Also, autosomal-dominant CMC was described in a family due to a lack of Th17 cells and cytokines, IL-17A and IL-22 [83]. Mutations in *IL-17A*, *IL-17RA*, *IL-17RC* and *ACT1* have been associated with CMC, and illustrate the importance of intact IL-17-signaling in antifungal immunity [80,84,85,86]. 

While rare, these genetic disorders lend persuasive evidence for the critical role of the Th17/IL-17 pathway in control of *Candida* infections especially at mucosal surfaces, and supports the concerns raised regarding the clinical use of anti-IL-17/IL-17R antibody therapies (see below) [65,87].

## 6. Anti-IL-17 Therapy in Autoimmunity and Increased Fungal Infection Risk

Th17-related proinflammatory cytokines IL-23 and IL-17 have been implicated in the pathogenic inflammation associated with autoimmunity [88]. Both IL-23 and IL-17 are the underlying pathogenic mechanisms of autoimmune diseases such as psoriasis, systemic lupus erythematosus (SLE) and rheumatoid arthritis (RA) [89]. Due to this, the IL-23/IL-17 pathway has been a target of therapeutic interventions for treatment of autoimmune diseases. 

Therapeutics targeting IL-17 specifically are currently being used or evaluated in clinical trials, and have proven especially efficacious for psoriatic conditions. Secukinumab (AIN457) and Ixekizumab (LY2439821), both humanized anti-IL-17A monoclonal antibodies have recently been approved by the United States Food and Drug Administration (FDA) for moderate-to-severe plaque psoriasis, psoriatic arthritis, as well as ankylosing spondylitis [90,91]. Moreover, there are additional antibodies that target IL-17RA with great therapeutic promise in psoriasis including Brodalumab (AMG 827) [92]. The potential off-target effects of neutralizing specific components of the IL-17 pathway in autoimmunity has been a point of concern when considering host immunity against fungal and bacterial infections [87]. Considering the essential role of IL-17/IL-17RA in antifungal immunity it is surprising that only mild cases of candidiasis have thus far been reported with Secukinumab treatment, yet patients on this or similar therapeutics should be monitored for fungal infections going forward [91].

## 7. Non-*albicans* Fungal Infections

The incidence of fungal infections caused by non-albicans *Candida* spp., as well as non-*Candida* spp., in both superficial as well as invasive forms of candidiasis, is significantly increasing (reviewed in [93]). *Aspergillus* spp. and *Candida* spp. constitute the majority of invasive fungal infections [93,94]. Non-albicans *Candida* spp. such as *C. tropicalis*, *C. parapsilosis* and *C. glabrata* are emerging as important causes of invasive candidiasis [9,95]. Other commonly reported non-albicans *Candida* species causing disease are *C. dubliniensis*, and *C. krusei* [9]. This shift in non-albicans *Candida* species causing invasive candidiasis is attributed to recent wide-spread use of antifungals targeting *C. albicans* [96], and is becoming more challenging due to the associated multi-drug resistance in species such as *C. glabrata* [97]. Moreover, some species are associated with specific malignancies more than others. For example, *C. glabrata* and *C. krusei* surpassed *C. albicans* as the leading cause of candidemia in patients with hematologic malignancies [98], and *C. parapsilosis* is frequently associated with the high mortality of invasive candidiasis in these patients [99]. Among *Candida* spp., and even between *C. albicans* strains, there are variations in cell wall composition, growth requirements and virulence factors which potentially affect the immune response initiated towards each (reviewed in [100]). Yet the various species can contain conserved cell wall epitopes. For example, a *C. albicans* epitope (ALS1/3 adhesin) stimulates distinct T cell responses, and in a cell culture system T cells responded to all *Candida* spp. (including *C. albicans*, *C. glabrata*, *C. krusei*, *C. tropicalis* and *C. dubliniensis*) by secreting IL-17 [101]. Even so, very little is known about the role of IL-17 in immunity to non-*C. albicans* species. IL-17 is dispensable for protection against disseminated *C. tropicalis* infection in mice, and rather CARD9 and TNF-α in neutrophils are essential [102]. *C. glabrata* is often co-isolated with *C. albicans* in OPC. While *C. glabrata* has been studied in systemic candidiasis models, it has been difficult to study in OPC models though, as it does not form hyphae or establish mucosal infection in mice. A recent study has shown that co-infection with *C. albicans* is required for *C. glabrata* to colonize and cause OPC [103]. Future studies using these types of co-infection models will help to elucidate if IL-17-related immune components are involved in protection to other *Candida* spp. 

## 8. Conclusions

Multiple findings in both humans and mice show the importance of IL-17 in protection against candidiasis. IL-17 is quickly induced upon *Candida* infection and in turn modulates pro-inflammatory cytokines, chemokines and antimicrobial proteins that protect against fungal infection via neutrophil influx and candidacidal activities [42]. Lack of effective vaccines and increasing drug-resistant fungal isolates are a challenge in the fight against the increasing incidence of fungal infections. The use of radiotherapy, chemotherapy, glucocorticoids and antibiotics, which all lead to an increased risk of candidiasis, is also growing. Moreover, targeting the IL-17 pathway as therapeutics for the treatment of autoimmune diseases such as psoriasis could exacerbate the problem of fungal infections. Therefore, a better understanding of IL-17-mediated immunity to *Candida* is even more compelling and necessary for the development of therapeutics that maintain antifungal immunity.
